# Pathological findings in South American camelids presented at a farm animal clinic in Northern Germany (2005–2021)

**DOI:** 10.1007/s11259-024-10369-1

**Published:** 2024-04-17

**Authors:** Saskia Neubert, Christina Puff, Sven Kleinschmidt, Patricia Kammeyer, Alexandra von Altrock, Michael Wendt, Matthias Gerhard Wagener

**Affiliations:** 1 Clinic for Swine and Small Ruminants, Forensic Medicine and Ambulatory Service, University of Veterinary Medicine Hannover, Foundation, 30173 Hannover, Germany; 2 Department of Pathology, University of Veterinary Medicine Hannover, Foundation, 30559 Hannover, Germany; 3 Lower Saxony State Office for Consumer Protection and Food Safety, Food and Veterinary Institute Braunschweig/Hannover, 30173 Hannover, Germany

**Keywords:** South American camelids, Llama, Alpaca, Necropsy, Diseases, Veterinary pathology

## Abstract

To expand the knowledge about common diseases in llamas and alpacas in Germany, a screening of the cases of South American camelids presented at the Clinic for Swine and Small Ruminants of the University of Veterinary Medicine Hannover, Germany from 2005 to the end of November 2021 was performed. A retrospective evaluation of necropsy reports from this period was conducted. Overall, necropsy reports were evaluated from 187 alpacas, 35 llamas and one vicuña (*n* = 223). A total of 50.2% of the dissected animals were thin or cachectic. Pathological alterations of the gastrointestinal tract were the most common findings (44.8%). In addition, liver changes were recorded, most frequently in adult animals. In contrast, diseases of the respiratory tract and the nervous system were found more frequently in juvenile animals. This study provides an overview of common pathologies in South American camelids in Germany and thus may help to recognise different disease symptoms at an early stage.

**Supplementary Information:**

The online version contains supplementary material available at 10.1007/s11259-024-10369-1.

## Introduction

Guanacos (*Lama guanicoe*) and vicuñas (*Vicugna vicugna)* as well as their domestic forms llamas (*Lama glama*) and alpacas (*Vicugna pacos*) are originally from South America. But especially the keeping of llamas and alpacas is also becoming more and more popular in Europe (Davis et al. 1998; Hengrave Burri et al. 2005; Neubert et al. 2021; Wagner et al. 2022). In an online survey of German alpaca and llama farms conducted in 2020, we found that more than half of those farms had started keeping South American camelids (SACs) in the previous six years, with alpacas being more popular than llamas. Endoparasitosis was mentioned as the most common cause of disease. The survey also showed that symptoms of diseases were rarely observed by owners (Neubert et al. 2021). This could be due to the fact that diseases were indeed rare, but it could also be assumed that disease symptoms were not accurately recognised by the owners since SACs are usually stoic animals and a dense fibre coat can mask a poor general condition (Van Saun 2009). Often, SACs presented at the clinic are found to be emaciated and anaemic without the owners having noticed this previously (Wagener et al. 2021b), so it is likely that pathological conditions in these animals will remain undetected.

In recent years, there have been several studies from different countries on the most common pathological changes found in alpacas and llamas. These include Canada (Shapiro et al. 2005), England and Wales (Twomey et al. 2014), the USA (Clarke and Breuer 2022; O’Conor Dowd 2014; Valentine and Martin 2007), Sweden (Björklund 2014, 2019) and Germany (Theuß et al. 2014). The study by Theuß et al. (2014) deals with necropsy material received at the University of Leipzig, and thus reflects the situation in the eastern parts of Germany. All the authors unanimously conclude that diseases of the gastrointestinal tract are most common in adult SACs. Emaciation was also frequently noted in these publications. Endoparasites, including mainly coccidia and gastrointestinal strongylids e.g. *Haemonchus contortus*, are particularly relevant in this context (Dubey 2018; Edwards et al. 2016; Franz et al. 2015). To our knowledge, no such information of necropsy material is yet available on the data from the northern and western parts of Germany. Furthermore, SACs in Germany are often kept as hobby animals and for use on trekking tours and therefore have close contact with humans (Neubert et al. 2021; Wagner et al. 2022). In this respect, zoonotic diseases play a major role in these animals. Various potential zoonoses such as infections with *Coxiella burnetii*, tuberculosis, sarcoptic mange and cryptosporidiosis have already been described in SACs (Halsby et al. 2017; Konieczny and Pomorska-Mól 2024; Rüfli et al. 2021). The aim of this study is therefore to present morphological findings of necropsies as well as results of further histological, microbiological and virological examinations of SACs from our clinic that is located in Northern Germany over a time span of 16 years.

## Materials and methods

A retrospective, longitudinal and observational study was carried out based on medical files of SACs that were presented at the Clinic for Swine and Small Ruminants, Forensic Medicine and Ambulatory Service of the University of Veterinary Medicine Hannover from January 2005 until the end of November 2021 and that were screened for necropsy reports including morphological, histological and further diagnostic results. The patient files of the animals were archived as paper files until August 2016. From then on, all files were archived digitally using “easyVET” (VetZ GmbH 2022). Relevant patient data were selected from these files and transferred to an Excel sheet (Microsoft Excel 2016) for data analyses. All data used in this study were collected during veterinary diagnostic procedures.

### Basic data on the animals

For each animal, the clinic identification, the species (alpaca/llama/vicuña/guanaco), the sex (female/male/castrated male), the age (juvenile: <1 year/adult: >1 year) and the year of necropsy were recorded. Additionally, it was documented if the animal had died spontaneously or if it had been euthanised.

### Animals at necropsy

A pathological examination was initiated and a necropsy report was available for 223 South American camelids, which included 187 alpacas (83.9%), 35 llamas (15.7%) and one vicuña (0.4%). No guanaco was dissected. Necropsy of 170 camelids (76.2%) was performed at the Department of Pathology of the University of Veterinary Medicine Hannover, and 53 animals (23.8%) were examined at the Food and Veterinary Institute, Lower Saxony State Office for Consumer Protection and Food Safety, Germany (LAVES). Only necropsy reports of whole carcasses were analysed. Examinations of individual organs were not assessed. The retrospective evaluation of the necropsy reports was conducted according to the following protocol (Neubert et al. 2022):

*Nutritional status*

The nutritional status was recorded according to the following scoring scheme:

0 = moderate or better: fat depots could be observed,

1 = thin: absence of body and subcutaneous fat depots,

2 = cachexia: serous atrophy of coronary fat and serous atrophy of bone marrow.

*Diagnoses*

Diagnoses were classified into the following organs or organ systems: cardiovascular system (heart, vessels); haematopoietic system (bone marrow, lymph nodes, spleen); respiratory system (nasal cavity and sinuses, larynx, trachea, lungs); body cavities (thoracic cavity, abdominal cavity, hernias); liver; genitourinary tract (reproductive organs, urinary organs); musculoskeletal system (bones, joints, muscles, tendons); skin; nervous system (brain and meninges, spinal cord, nerves); eyes or ears. The changes in the gastrointestinal tract were assigned even more precisely to a specific region of the gastrointestinal tract. This involved a classification into mouth (teeth, jaws, oral cavity), oesophagus, compartment system and intestine. Furthermore, the compartment system was examined for the presence of ulcerations. The alterations were divided into the scores: 0 = no erosions or ulcerations, 1 = erosion(s) (as a preliminary stage of ulceration), 2 = ulceration(s), and 3 = perforated gastric ulcer(s). In addition, for animals with gastric erosions and ulcerations, the compartment or compartments most severely affected were recorded.

A scoring system was used to systematically assess the clinical relevance of the pathological changes in the affected organs or organ systems:

0 = no findings or findings without clinical relevance,

1 = minor or questionable clinical relevance,

2 = clinically relevant findings.

In addition, a categorisation of pathological changes (malformation, degeneration, inflammation, tumour, circulatory disorder, other) was performed. In case of multiple changes, the organ was assigned to the predominant category. Diagnoses interpreted as agonal changes (such as lung congestion) and body cavity effusions due to cachexia were not recorded. Furthermore, only those lymph node changes were recorded that affected several lymph nodes in different regions of the body (instead of changes in individual lymph nodes due to inflammation in the tributary area). The detection of gastrointestinal parasites during necropsy was also listed using a score of 0 (no detection), 1 (low-degree detection of minor clinical relevance) and 2 (medium- to high-degree detection of clinical relevance). In addition, general diagnoses [anaemia, sepsis, systemic mineralisation, systemic mycosis, cachexia and uraemia (diagnosed by examination of the aqueous humour for urea concentration)] were noted as free text. Only a urea concentration of ≥ 180 mg/dL (physiological reference value is < 50 mg/dL) was rated as uraemia in the general diagnoses. The diagnosis of anaemia was based on the morphological appearance during necropsy and was independent of laboratory investigations. The results of additional microbiological and virological examinations were recorded.

## Results

**Fig. 1:**
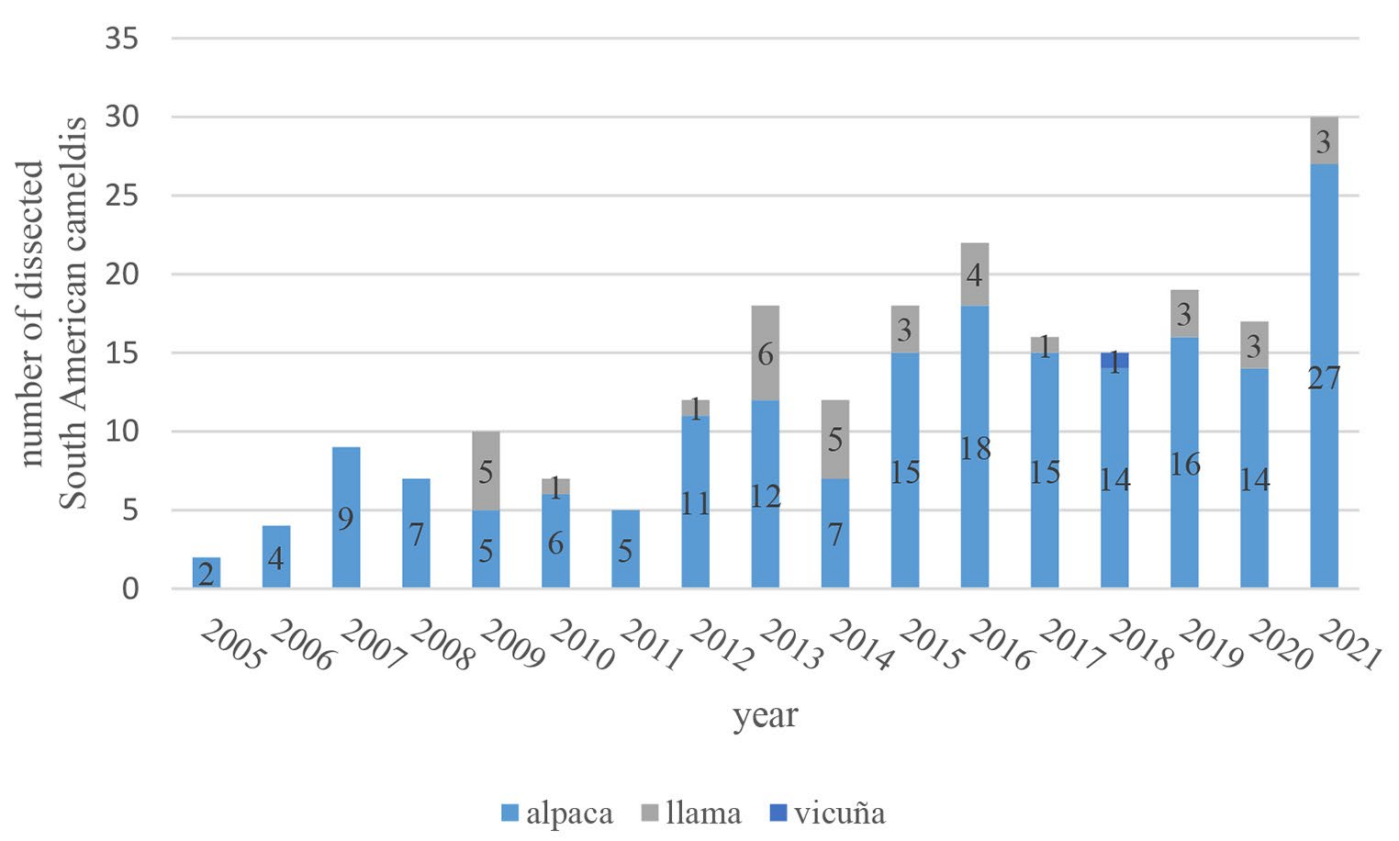
Number of dissected South American camelids (SACs) from the Clinic for Swine and Small Ruminants, Forensic Medicine and Ambulatory Service of the University of Veterinary Medicine Hannover, Germany per year. 2021 includes data only until the end of November

Overall, the number of dissected SACs in the studied period increased (Fig. 1).

**Fig. 2:**
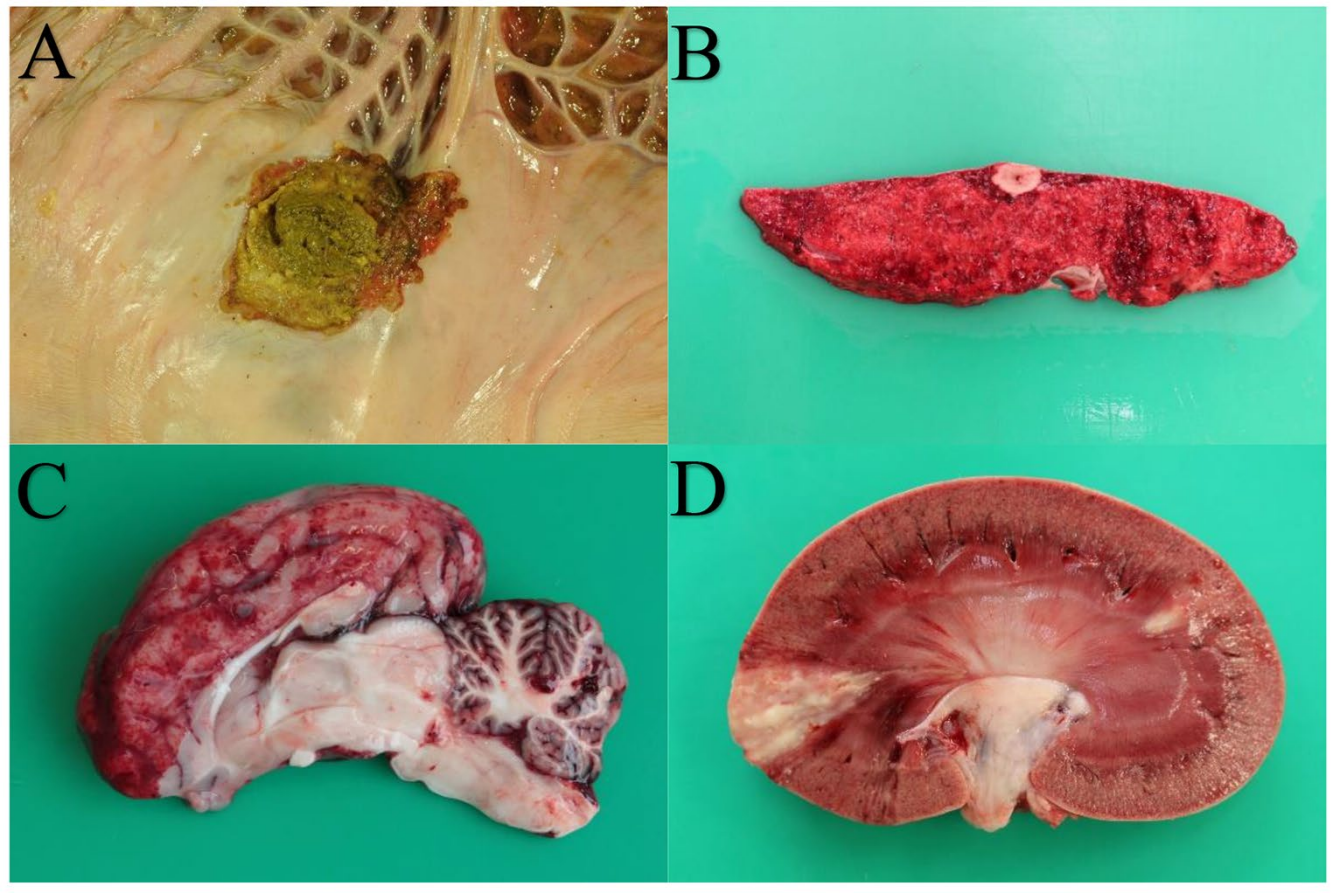
Selected pictures of pathological findings in South American camelids. **A** – Gastric squamous cell carcinoma (C1). **B** – Mycotic, granulomatous pneumonia. **C** – Haemorrhagic, partly suppurative encephalomyelitis with detection of *Escherichia coli*. **D** – Renal infarctions with detection of fungal hyphae (*Candida albicans*)

More females than males and more adult than juvenile SACs underwent necropsy. An overview of the age and sex of the dissected animals, summarised for the three animal species, is displayed in Table 1.

**Table 1:** Age and sex distribution of the dissected animals, summarised for alpaca, llama and vicuña (*n* = 223)

Age	Female	Male	Castrated male	Total
**Juvenile (< 1 year)**	38 (17.0%)	31 (13.9%)	0 (0%)	69 (30.9%)
**Adult (> 1 year)**	87 (39.0%)	39 (17.5%)	28 (12.6%)	154 (69.1%)
**Total**	125 (56.1%)	70 (31.4%)	28 (12.6%)	223 (100.0%)

About half of the animals (50.2%; *n* = 112) were thin or cachectic (scores 1 and 2) at necropsy. Cachexia was diagnosed in a total of 75 of the 223 animals (33.6%). More than half of the SACs (54.7%; *n* = 122) had at least one general diagnosis at necropsy. In addition, uraemia (urea concentration in the aqueous humour ≥ 180 mg/dL) was recorded in 42 animals (18.8%) and anaemia in 24 animals (10.8%). Less frequently, the carcasses revealed systemic mineralisation due to vitamin D intoxication (3.1%; *n* = 7 from five different herds) or manifestations of septicaemia (3.1%; *n* = 7). Systemic mycosis was found in two llamas and one alpaca (1.3%; twice Candida species and once not further specified). Plant intoxication was suspected in four animals (oleander (*n* = 3) and rhododendron (*n* = 1)).

The gastrointestinal tract was the organ system most frequently affected by pathological changes of clinical relevance (44.8% of all examined animals (*n* = 100)), in both young and adult animals. In terms of frequency, pathological changes in the liver (mostly adult animals) and respiratory tract (mostly juvenile animals) followed. In addition, alterations of the nervous system (especially meningitis and encephalitis) played a more important role in juvenile animals than in adults. A detailed overview of the frequency of pathological changes in the different organs and organ systems, divided into juvenile and adult animals, is provided in Table 2.

**Table 2:** Frequency of clinically relevant affected organs/organ systems in the dissected South American camelids, divided into juvenile (< 1 year) and adult (> 1 year) animals (*n* = 223). Many animals had pathological changes in multiple organs

Affected organ/ organ system	Adult (*n* = 154)	% of adults	Juvenile (*n* = 69)	% of juveniles	Total (*n* = 223)	Total in %
**Gastrointestinal tract**	75	48.7%	25	36.2%	**100**	**44.8%**
**Liver**	48	31.2%	10	14.5%	**58**	**26.0%**
**Respiratory system**	32	20.8%	20	29.0%	**52**	**23.3%**
**Body cavities**	33	21.4%	13	18.8%	**46**	**20.6%**
**Genitourinary tract**	22	14.3%	10	14.5%	**32**	**14.3%**
**Nervous system**	10	6.5%	15	21.7%	**25**	**11.2%**
**Cardiovascular system**	14	9.1%	10	14.5%	**24**	**10.8%**
**Haematopoietic system**	16	10.4%	2	2.9%	**18**	**8.1%**
**Musculoskeletal system**	10	6.5%	3	4.3%	**13**	**5.8%**
**Skin**	12	7.8%	1	1.4%	**13**	**5.8%**
**Eyes/ears**	2	1.3%	1	1.4%	**3**	**1.3%**

Among the 100 SACs with changes in the gastrointestinal tract, most findings were in the compartments (65.0%) and the intestine (40.0%). Pathological changes in the oral cavity (17.0%) and the oesophagus (12.0%) were less common.

In general, partly erosive, partly ulcerative changes (scores 1, 2 and 3) were detected in the compartments of almost one third of the animals (30.5%; *n* = 68). Ulcerations (scores 2 and 3) were detected in nine out of 35 llamas (25.7%) and 44 out of 187 alpacas (23.5%), with perforated ulcerations found exclusively in alpacas. Including the animals with erosions (scores 1, 2 and 3), the proportion of affected llamas (40.0%; *n* = 14/35) and alpacas (28.9%; *n* = 54/187) increased even more significantly. The majority of erosive and/or ulcerative lesions were found in compartment 3 (C3) (69.1%). Detailed information about gastric ulcers in those alpacas has been recently published by the authors (Neubert et al. 2022).

In most animals, no endoparasites could be detected (76.2%; *n* = 170). Thirty-six findings (16.1%) were classified as low-degree infestation, 17 animals had high-degree endoparasitosis (7.6%).

In the liver and urogenital tract, degenerative changes predominated. In particular, hepatic lipidoses and centrolobular hepatocellular necrosis were found. In the genitourinary tract, degenerative changes of the kidneys characterised by nephrosis with mineralisation were particularly frequent. Table 3 lists the diagnoses found in the respective organs/organ systems grouped in categories of pathological changes.

**Table 3:** Diagnoses found in the respective organs/organ systems in the dissected South American camelids (*n* = 223) grouped in categories of pathological changes, indicated as number of animals and percentage of the category in the respective organ system. Many animals had pathological changes in multiple organs

Affected organ / organ system	Categorisation of pathological lesions	Diagnosis	Frequency (*n* = 223)
Gastrointestinal tract: mouth (*n* = 17)	Malformation (17.6%)	Malformation of the jaw	2
		Cleft palate	1
	Inflammation (70.6%)	Stomatitis/glossitis	5
		Alveolitis/tooth root infection/osteomyelitis (one case with stomatitis)	7
	Other (11.8%)	Mucosal ulcers	2
Gastrointestinal tract: oesophagus (*n* = 12)	Degeneration (8.3%)	Muscular caliber variation	1
	Inflammation (91.7%)	Oesophagitis	11
Gastrointestinal tract: compartments (*n* = 65)	Degeneration (4.6%)	Mineralisation (due to vitamin D intoxication; two cases additionally with ulceration)	3
	Inflammation (81.5%)	Gastritis (eight cases with detection of fungal hyphae)	53
	Tumour (10.8%)	Adenocarcinoma	3
		Squamous cell carcinoma	3
		Tumour metastases (malignant blastoma)	1
	Other (3.1%)	Acidosis C1	2
Gastrointestinal tract: intestine (*n* = 40)	Inflammation (90.0%)	Enteritis/colitis/enterocolitis (six cases with perforation; two cases with detection of fungal hyphae)	36
	Tumour (7.5%)	Squamous cell carcinoma	1
		Tumour metastases (malignant blastoma; lymphoma)	2
	Circulatory disorder (2.5%)	Volvulus with jejunitis	1
Liver (*n* = 58)	Degeneration (58.6%)	Hepatic necrosis	23
		Hepatic lipidosis	8
		Liver cirrhosis	2
		Liver cell vacuolation	1
	Inflammation (34.5%)	Hepatitis (two cases with detection of fungal hyphae)	20
	Tumour (6.9%)	Round cell tumour	1
		Tumour metastases (2x adenocarcinoma; 1x squamous cell carcinoma)	3
Respiratory system (*n* = 52)	Malformation (5.8%)	Choanal atresia	2
		Absent lung lobe	1
	Degeneration (19.2%)	Pulmonary mineralisation (vitamin D intoxication in five cases)	6
		Pulmonary fibrosis (one case additionally with mineralisation due to vitamin D intoxication)	4
	Inflammation (61.5%)	Pneumonia (mainly purulent; suspected aspiration pneumonia in three cases; one case with purulent rhinitis)	26
		Pneumonia with detection of fungal hyphae	3
		Purulent rhinitis (one case with osteolysis and detection of fungal hyphae)	2
		Purulent laryngitis with pleuropneumonia	1
	Tumour (3.8%)	Tumour metastases (squamous cell carcinoma)	2
	Circulatory disorder (5.8%)	Haemorrhages (one case with pneumonia)	2
		Acute respiratory distress syndrome	1
	Other (3.8%)	Atelectasis	2
Body cavities (*n* = 46)	Malformation (4.3%)	Diaphragmatic hernia (one case unclear if congenital)	2
	Inflammation (73.9%)	Peritonitis (one case with pleuritis and pericarditis, two cases with omphalitis/omphaloarteritis)	24
		Pleuritis	3
		Local serositis of the intestine	2
		Omphalitis	1
		Transmural inflammation of the abdominal wall	1
		Inflammation of the diaphragm/mesentery (one case with detection of fungal hyphae)	3
	Tumour (10.9%)	Tumour metastases (2x adenocarcinoma; 2x squamous cell carcinoma; 1x malignant blastoma with peritonitis)	5
	Circulatory disorder (8.7%)	Hydrothorax (one case with hydropericard and hydroperitoneum)	3
		Hydroperitoneum	1
	Other (2.2%)	Uroperitoneum	1
Genitourinary tract (*n* = 32)	Degeneration (46.9%)	Renal mineralisation (vitamin D intoxication in six cases)	7
		Nephrosis (one case additionally with endometritis)	4
		Non-specific nephropathy	3
		Renal fibrosis	1
	Inflammation (40.6%)	Nephritis (four cases with detection of fungal hyphae; one case additionally with fungal cystitis; one case additionally with endometritis)	12
		Endometritis	1
	Tumour (3.1%)	Malignant blastoma	1
	Circulatory disorder (3.1%)	Uterine haematoma	1
	Other (6.3%)	Ruptured urinary bladder	2
Nervous system (*n* = 25)	Malformation (8.0%)	Aplasia of the olfactory bulb	2
	Degeneration (12.0%)	Laminar cerebrocortical necrosis	3
	Inflammation (56.0%)	Encephalitis and/or meningitis (three cases with detection of fungal hyphae; one case with detection of *Listeria monocytogenes*; one case with detection of *Escherichia coli*)	12
		Brain abscess (cerebellum; pituitary gland)	2
	Circulatory disorder (16.0%)	Brain haemorrhage	4
	Other (8.0%)	Laceration spinal cord	1
		Cerebrospinal nematodiasis (with encephalomyelomalacia and meningoencephalomyelitis)	1
Cardiovascular system (*n* = 24)	Malformation (4.2%)	Defect of the right atrium	1
	Degeneration (29.2%)	Myocardial degeneration/necrosis	3
		Myocardial fibrosis	1
		Myocardial mineralisation (due to vitamin D intoxication)	3
	Inflammation (58.3%)	Endocarditis	1
		Myocarditis (one case with detection of fungal hyphae)	7
		Myocarditis and pericarditis (with detection of fungal hyphae)	1
		Pericarditis and/or epicarditis	5
	Tumour (8.3%)	Tumour metastases (lymphoma; squamous cell carcinoma)	2
Haematopoietic system (*n* = 18)	Degeneration (5.6%)	Splenic necrosis	1
	Inflammation (38.9%)	Splenitis	2
		Lymphadenitis (one case additionally with splenitis)	3
		Lymphadenitis with detection of fungal hyphae (one case additionally with splenitis)	2
	Tumour (44.4%)	Lymphoma Round cell tumour	1 1
		Tumour metastases (3x squamous cell carcinoma, 2x adenocarcinoma; 1x malignant blastoma)	6
	Other (11.1%)	Generalised spleen and lymph node depletion	2
Musculoskeletal system (*n* = 13)	Malformation (7.7%)	Angular limb deformity	1
	Degeneration (30.8%)	Muscular degeneration	3
		Degenerative joint disease (after chronic patellar luxation)	1
	Inflammation (23.1%)	Arthritis/myositis	2
		Osteomyelitis	1
	Other (38.5%)	Fractures (2x femur; 1x spine)	3
		Rickets	1
		Hip dislocation	1
Skin (*n* = 13)	Inflammation (100.0%)	Dermatitis/hyperkeratosis (five cases with detection of mange; two cases with detection of fungal hyphae)	10
		Local dermatitis	2
		Abscess	1
Eyes/Ears (*n* = 3)	Inflammation (100.0%)	Iridocyclitis and retinitis with hypopyon	1
		Endophthalmitis with retinal detachment	1
		Osteomyelitis inner ear	1

The complete frequency of affected organ systems, divided into score 0 (no findings or findings without clinical relevance), score 1 (diagnoses of minor or questionable clinical relevance) and score 2 (clinically relevant findings) can be found in the Supplementary Material (S1).

Microbiological examination of pathomorphologically altered organs was performed only in individual cases. Clostridia were detected in the intestines of nine animals by culture, in seven cases these were associated with enteritis or enterocolitis. The clostridia were differentiated in six animals; all were *Clostridium perfringens* type A infestations. Clostridial toxin was detected in two of these animals. In the respiratory tract, especially in the lungs, various pathogens were detected in 10 animals. The pathogens *Staphylococcus aureus*, *Streptococcus* spp., *Trueperella pyogenes*, *Proteus* spp. and *Escherichia coli* were involved. *Escherichia coli* was isolated in the brain of three animals, in one animal with inflammatory signs in the nervous system, and Listeria was detected by microbiological examination of brain tissue. In addition, fungal spores (*Candida albicans*, *Mucor* spp., *Aspergillus* spp.) were isolated in various organs in individual cases. Most of the animals were tested for Bluetongue virus (BTV); in case of suspicion, other viruses (e.g. Schmallenberg virus (SBV); bovine viral diarrhoea virus (BVD)) were also included. No viral infections were detected in the tested animals.

Malignant neoplasms were rare (4.0%; *n* = 9): Adenocarcinomas (*n* = 3) and squamous cell carcinomas (*n* = 3) of the stomach were the most common neoplasia. Lymphoma, round cell tumour and malignant blastoma were detected in one animal each. SACs over 10 years of age were mainly affected (*n* = 6); the round cell tumour was diagnosed in a young animal (under one year of age).

Malformations were also recorded in the study material (5.8%; *n* = 13). Clinically relevant alterations were choanal atresia with absence of the *bulbi olfactorii* in two animals. Two SACs showed major jaw abnormalities, in one animal additionally in combination with a heart malformation. Furthermore, one animal each was found to have a cleft palate, dysplasia of the limb skeleton and a diaphragmatic hernia in combination with a missing lobe of the lung. In the remaining six animals, malformations of minor clinical relevance were recorded (low-degree umbilical hernia in three animals, including one associated with a narrow cleft palate; in one animal each, a unilateral, a grade 1 patellar luxation, a cleft lip, or unilateral absence of kidney and uterine horn). Examples of pathological findings are presented in Fig. 2.

## Discussion

The present data on necropsy reports seem to indicate the trend of an increasing number of South American camelids (SACs) kept in Europe.

Of the 223 dissected animals, 50.2% had a poor nutritional status and 33.6% were even diagnosed with cachexia. Also, a previously published retrospective evaluation of patient data from the Clinic for Swine and Small Ruminants, University of Veterinary Medicine Hannover revealed that 60% of the hospitalised alpacas and 70% of the hospitalised llamas had a body condition score (BCS) below 3 (a BCS of 3 is considered optimal (Wagener and Ganter 2020)). In this context, a reduced BCS was often associated with anaemia and shifts in white blood count (Wagener et al. 2021b). In other evaluations of necropsy reports of SACs, emaciation was also frequently noted (Björklund et al. 2019; Clarke and Breuer 2022; Theuß et al. 2014). In the clinic and especially at necropsy, there are animals with various diseases that can explain the poor nutritional status. However, it is often not possible to determine from the pathological examination whether these findings were the cause of the cachexia or whether the cachexia was caused by other factors such as incorrect or insufficient diet or other stress factors. According to the literature, common causes for a low BCS in SACs are chronic infectious disease, endoparasites, dental problems and nutrition (Wagener et al. 2023a). Nevertheless, with the large number of emaciated animals, the question remains whether emaciation of animals in the flock is often not recognised or recognised too late by the owners. In order to detect emaciation as a possible sign of disease processes in time, regular recording of the BCS is recommended (Gauly and Vaughan 2018; Wagener and Ganter 2020).

According to several studies, diseases of the gastrointestinal tract, including high incidence of endoparasites, are most common in SACs compared to diseases of other organ systems (Björklund et al. 2019; Clarke and Breuer 2022; O’Conor Dowd 2014; Shapiro et al. 2005; Theuß et al. 2014; Twomey et al. 2014). Gastric erosions and ulcerations are also a common finding in this context (Fowler 2010b; Hund and Wittek 2018; Neubert et al. 2022; Smith et al. 1994; Theuß et al. 2014). In line with these studies, the gastrointestinal tract was also most frequently affected by clinically relevant pathological changes in both juvenile and adult SACs in the present investigation. Especially gastric ulcers regularly occurred in the dissected animals. Previous studies have already described the major relevance of gastric ulcers in SACs (Hund and Wittek 2018; Smith et al. 1994), which is confirmed by our results. A detailed evaluation of these alpacas with gastric ulcers has been recently published by the authors. It would appear that concomitant diseases are a promoting factor for the occurrence of gastric ulcers (Neubert et al. 2022). Carvallo et al. (2020) found gastric ulcers to be associated with *Fusobacterium necrophorum.* However, as no microbiological examinations of the gastric ulcers were carried out in this study, no statement can be made about the significance of this bacterium in these cases. Furthermore, tooth root infections, sometimes with osteomyelitis of the surrounding bone, seem to be a common problem of the gastrointestinal tract in SACs (Anderson 2006). The premolars and molars are particularly affected (Niehaus and Anderson 2007). In the present study, such infections were occasionally observed (Table 3). The gastroenteritis cases found in other studies were frequently caused by parasites (Björklund et al. 2019; Theuß et al. 2014; Twomey et al. 2014). In contrast to these studies, infestation with endoparasites, which was assessed separately from the digestive tract in the present study, was seldom (23.8%) at necropsy. But it must be kept in mind that many animals had been previously treated in the clinic and had therefore often been dewormed during the clinic stay if endoparasites had been found in the parasitological faecal examination. Thus, the contribution of parasites to the cases of gastroenteritis cannot be ruled out in our study. Other infectious agents associated with gastroenteritis in SACs are predominantly *Clostridium* spp., *Escherichia coli* and *Fusobacterium necrophorum* (Clarke and Breuer 2022). However, possible pathogens could only be detected sporadically in this study.

In addition to diseases of the gastrointestinal tract, diseases of the respiratory system seem to be of great relevance (Theuß et al. 2014). Pathological changes of the respiratory tract were recorded in 23.3% of all dissected animals. Common bacterial pathogens are the same as those found in other farm animals. In particular, opportunistic bacteria seem to be frequent causative pathogens (Fowler 2010e). Although aspiration pneumonia was suspected in three animals, the aetiology and pathogenesis of the pneumonia could not be clarified in most cases. Pneumonia can be caused in particular by airborne infection or haematogenous dissemination from other disease processes (Cebra 2014). Opportunistic pathogens only lead to disease when the general condition of the animal is reduced. A possible explanation for this could be that many animals at necropsy were already in a very poor general condition. A primarily viral infection with secondary bacterial colonisation should also be considered, as hardly any virological examinations were conducted. However, these results are in contrast to studies by other authors, in which alterations of the respiratory tract were found with a much lesser frequency (Björklund et al. 2019; Clarke and Breuer 2022; O’Conor Dowd 2014).

The high proportion of pathological alterations in the liver and body cavities in the present study also complies with the findings of other authors. According to Shapiro et al. (2005), hepatic disease was the third most common diagnosis in SACs over one year of age. In a retrospective review of necropsy reports of camelids, Björklund (2014) regularly observed hepatic disorders, mainly as hepatic lipidosis or liver fluke infestation. In the present study, liver alterations also manifested predominantly as degenerative changes. Hepatic lipidoses were frequently observed in the present study, consistent with the findings of Björklund (2014). Hypoxic degeneration secondary to anaemia may be considered as the cause of hepatocellular necrosis that was often noted in the dissected animals. In a retrospective study, it was found that around 50% of the SACs admitted to our clinic had a packed cell volume (PCV) below the reference range (Wagener et al. 2021b). Common causes of anaemia in llamas and alpacas are blood loss, mainly due to *Haemonchus contortus*, as well as trace element deficiencies and cachexia (Wagener et al. 2023b). Anaemia is associated with poor nutritional status in most SACs (Wagener et al. 2021b). The high incidence of animals with pathological changes in the body cavities should be interpreted with caution because many of these changes usually resulted from pathological processes in other organs and organ systems. For example, peritonitis was diagnosed in animals with perforations in the gastrointestinal tract.

Pathological changes of the nervous system were diagnosed in a total of 11.2% of all SACs, whereby mainly juvenile animals were affected. In line with this, Twomey et al. (2014) primarily recorded bacterial meningitis or meningoencephalitis in crias at necropsy. They suspected that the occurrence of these cases were also likely due to neonatal management problems (Twomey et al. 2014). In turn, Shapiro et al. (2005) listed neurological disease as the second most common diagnosis in SACs over one year of age. In the present study three animals were found to have cerebral degeneration, the microscopic lesions were consistent with those of polioencephalomalacia. Polioencephalomalacia is a common metabolic disorder of ruminants due to thiamine deficiency, often resulting from rapid dietary changes and excessive carbohydrate ingestion and therefore the growth of thiaminase producing bacteria (Bedenice and Whitehead 2016). Reduced feed intake, the ingestion of plants containing thiaminase or decreased intestinal absorption can also lead to this condition (Beck et al. 1996; Himsworth 2008). Other authors have also diagnosed cerebrocortical necrosis in pathological examinations of SACs (Beck et al. 1996; Björklund et al. 2019; Himsworth 2008; Kiupel et al. 2003; Twomey et al. 2014). The underlying cause can often not be clarified, which was also the case in the three animals in this study.

According to the present study, skin lesions and ectoparasites seem to be a rare problem in SACs. However, both the literature and our own clinical experience give a different impression; ectoparasites, especially mange mites, are very common in SACs. According to existing studies, prevalences of *Chorioptes* sp. range from 40 to 50% in SAC populations (D’Alterio et al. 2005; Schlögl et al. 2010). Also in the Clinic for Swine and Small Ruminants, University of Veterinary Medicine Hannover, mange mites are regularly detected in SACs. Examination of 165 skin scrapings from the period of 2008 to 2021 revealed positive findings, particularly Chorioptes mange, in 122 animals (73.9%, data not presented). However, according to preliminary reports, owners noticed itching or skin lesions in their animals only in exceptional cases (*n* = 22; 18%). Even though these data are not representative, they suggest that mange often goes unrecognised by animal owners and only becomes noticeable when generalised changes occur. In addition, it must be taken into account that histological examinations for ectoparasites were not regularly performed at necropsy.

Eye and ear diseases do not seem to play an important role in SACs. Nonetheless, these organs are not routinely examined histologically at necropsy unless there is macroscopic or pre-report evidence.

Vitamin D intoxication resulting in generalised mineralisation was found in seven examined animals. In SACs in Europe, vitamin D has to be supplemented due to the lower UV exposure than in the Andes, as no sufficient synthesis can take place in the skin of the animals. Deficiency can lead to calcium/phosphorus imbalances and rickets in growing animals (Van Saun 2014). However, it is crucial to avoid overdosing, since the overdosed vitamin is not excreted and subsequently leads to life-threatening systemic mineralisation (Fowler 2010c), as in the seven animals in the present study. The findings of some of these animals have already been described in detailed case reports (Helmer et al. 2017; Wagener et al. 2021a).

Renal mineralisation due to vitamin D intoxication as well as other renal disorders, for example nephritis, can lead to renal uraemia. This might explain the increased urea concentration in the aqueous humour of some animals. At the same time, in some animals without kidney lesions, no exact cause for the uraemia could be determined. Possible prerenal causes of uraemia include sepsis or dehydration (Russell and Roussel 2007).

Further microbiological diagnostics were only carried out on a very small number of animals, so the results of the present study are not very representative. Since a large proportion of the SACs were pretreated with antibiotics, falsified results must also be taken into account, which further limits the significance of the results. Overall, however, the detected pathogen species are in line with the results of Theuß et al. (2014). Clostridia in particular seem to be of importance in SACs (Fowler 2010d). Various *Clostridium* spp. can cause many different diseases in SACs as well as other animal species. A common pathogen for enterotoxaemia is *Clostridium perfringens*, of which types A, B, C, D and E can be classified. Their virulence is characterised by various toxins produced by the clostridia. Affected animals may suffer from diarrhoea, colic, enteritis and sudden death (Konieczny and Pomorska-Mól 2024). Although clostridia were detected in the intestines of nine animals in this study, toxins were only detected in two animals. As *Clostridium perfringens* is part of the normal intestinal microflora (Fowler 2010d), in most of the cases no definite statement can be made as to whether the Clostridia caused the disease in these animals. Fungal hyphae were detected in various organs, three animals even had systemic mycosis. A few case reports on systemic or local mycosis in SACs have already been published; the respiratory tract and lesions of the skin or in the gastrointestinal tract, e.g. gastric ulcers, are described as portals of entry. Haematogenous spread of fungal infection may occur, especially in immunosuppressed animals (Bildfell et al. 2002; Hughes and Mueller 2008; Kramer et al. 2008; Severo et al. 1989). Only occasionally were virological examinations conducted, mainly for Bluetongue virus (BTV), and less frequently for rabies, bovine viral diarrhoea virus (BVD) or Schmallenberg virus (SBV) based on clinical suspicion. Although these examinations did not reveal any positive result, case reports about viral infections like BTV (Allen et al. 2015; Ortega et al. 2010), BVD (Belknap et al. 2000; Foster et al. 2007) or Borna virus (Schulze et al. 2020) in SACs can be found in the literature. Theuß et al. (2014) detected in their retrospective study of SAC necropsies ovine herpesvirus-2 (malignant catarrhal fever) and orthopoxvirus in two and three alpacas, respectively. Since only a small proportion of animals were virologically tested in the present study, the negative results need to be interpreted with caution.

Malignant neoplasms were found with a prevalence of 4.0% in the dissected animals. Theuß et al. (2014) came to a similar conclusion with a determined prevalence of 3.0% after analysing necropsy reports of SACs. In contrast, other studies recorded slightly higher prevalences ranging from 5.2% up to 8.8% (Aboellail et al. 2021; Björklund 2014; Clarke and Breuer 2022; O’Conor Dowd 2014; Valentine and Martin 2007). According to the literature, Lymphomas are one of the most commonly diagnosed neoplasia in SACs, often occurring in young animals under five years of age (Aboellail et al. 2021; Bildfell et al. 2012; Zanolari et al. 2018). In contrast, in the present study, adenocarcinomas and squamous cell carcinomas represented the most common neoplasia and predominantly affected animals over 10 years of age.

Various malformations occurring in SACs, such as limb deformities, polydactyly or cranial deformities, have already been described in the literature (Fowler 2010a; Leipold et al. 1994; Zanolari et al. 2018). Congenital defects seem to be more common in camelids than in other farm animals (Fowler 2010a). In this respect, the relatively large proportion of malformations recorded in our study (5.8%) is in line with the current literature. Two animals revealed choanal atresia. Studies indicate that this malformation is relatively common in llamas and alpacas (Bertin et al. 2015; Reed et al. 2010; Zanolari et al. 2018). In American llama populations, it accounts for approximately 10% of all congenital defects (Leipold et al. 1994).

In summary, SACs seem to be most commonly affected by pathologies of the gastrointestinal tract. Gastroenteritis in particular, including gastric ulcers, occured frequently. At the same time, a large number of animals suffered from emaciation. Regular monitoring of the BCS is therefore of major importance. The results of the study may help to define the common pathological patterns of SACs and thus help veterinarians and animal owners to recognise diseases at an early stage. However, the retrospective format and relatively small sample size must be considered as limitations of the study.

These results have already been published in German as part of the author’s thesis (Neubert 2022).

### Electronic supplementary material

Below is the link to the electronic supplementary material.

Supplementary Material 1

## Data Availability

The datasets generated in this study are included in this article (and its supplementary information file) and are also available from the corresponding author upon reasonable request.
