# Supplementary material for: Pathological findings in South American camelids presented at a farm animal clinic in Northern Germany (2005–2021)
Source: Vet Res Commun. 2024 Apr 17;48(4):2121–34. doi: 10.1007/s11259-024-10369-1 (PMC11315760; doi:10.1007/s11259-024-10369-1)
Supplement: Supplementary file 1 — Supplementary Material 1 [file 11259_2024_10369_MOESM1_ESM.pdf]

Online Resource 1

Pathological findings in South American camelids presented at a farm animal clinic in Northern Germany (2005 – 2021)

Saskia Neubert (0000-0002-5602-6686)[1,*], Christina Puff (0000-0002-2592-6868)[2], Sven Kleinschmidt (0000-0003-2690-6153)[3], Patricia Kammeyer[3], Alexandra von Altrock (0000-0001-7507-3608)[1], Michael Wendt (0000-0002-3545-0859)[1] and Matthias Gerhard Wagener (0000-0003-3366-8579)[1]

[1]Clinic for Swine and Small Ruminants, Forensic Medicine and Ambulatory Service, University of Veterinary Medicine Hannover, Foundation, 30173 Hannover, Germany

[2]Department of Pathology, University of Veterinary Medicine Hannover, Foundation, 30559 Hannover, Germany

[3]Lower Saxony State Office for Consumer Protection and Food Safety, Food and Veterinary Institute Braunschweig/Hannover, 30173 Hannover, Germany

*Correspondence: neubert.saskia@web.de

S1. Frequency of affected organs/organ systems in dissected South American camelids, divided into score 0 (no findings or findings without clinical relevance), score 1 (diagnoses of minor or questionable clinical relevance) and score 2 (clinically relevant findings) (*n*=223).

Organ/organ system	Score 0	% of total	Score 1	% of total	Score 2	% of total
**Cardiovascular system**	179	80.3%	20	9.0%	24	10.8%
**Haematopoietic system**	203	91.0%	2	0.9%	18	8.1%
**Respiratory system**	119	53.4%	52	23.3%	52	23.3%
**Gastrointestinal tract**	66	29.6%	57	25.6%	100	44.8%
**Body cavities**	154	69.1%	23	10.3%	46	20.6%
**Liver**	123	55.2%	42	18.8%	58	26.0%
**Genitourinary tract**	157	70.4%	34	15.2%	32	14.3%
**Musculoskeletal system**	192	86.1%	18	8.1%	13	5.8%
**Skin**	192	86.1%	18	8.1%	13	5.8%
**Nervous system**	179	80.3%	19	8.5%	25	11.2%
**Eyes/ears**	214	96.0%	6	2.7%	3	1.3%
